# Dysregulated Repair in Aging and Disease: Extracellular Vesicles as an Emerging Protective Strategy

**DOI:** 10.3390/cells15080662

**Published:** 2026-04-09

**Authors:** Anna Calabrò, Giulia Accardi, Alexander Batista-Duharte, Mattia Emanuela Ligotti, Alejandra Pera, Chiara Puleo, Maurizio Soresi, Giuseppina Candore, Anna Aiello

**Affiliations:** 1 Laboratory of Immunopathology and Immunosenescence, Department of Biomedicine, Neuroscience and Advanced Diagnostic, University of Palermo, 90134 Palermo, Italy; anna.calabro@unipa.it (A.C.); giulia.accardi@unipa.it (G.A.); chiara.puleo@unipa.it (C.P.); anna.aiello@unipa.it (A.A.); 2 Molecular Immunology CTS-208 Group, Cell Biology, Physiology and Immunology Department, University of Córdoba, 14004 Córdoba, Spain; bc2badua@uco.es (A.B.-D.); h02peroa@uco.es (A.P.); 3 Immunology and Allergy GC01 Group, Maimonides Biomedical Research Institute of Córdoba, 14004 Córdoba, Spain; 4 Department of Research, IRCCS-ISMETT (Istituto Mediterraneo per i Trapianti e Terapie ad Alta Specializzazione), 90127 Palermo, Italy; mligotti@ismett.edu; 5 Department of Health Promotion Sciences, Maternal and Infant Care, Internal Medicine and Medical Specialties (PROMISE), University of Palermo, 90127 Palermo, Italy; maurizio.soresi@unipa.it

**Keywords:** tissue repair, healing, immune system, immunosenescence, ulceration, fibrosis, wound healing–fibrosis–cancer, extracellular vesicles, regenerative medicine

## Abstract

Tissue repair is a finely organized process that progresses via a series of phases, including hemostasis, inflammation, proliferation, and remodeling, which are coordinated by immune–stromal interactions. Aging profoundly dysregulates these processes through mechanisms such as immunosenescence and inflammaging, cellular senescence, chronic inflammation, and extracellular matrix alterations, ultimately contributing to typical age-related progression. This review discusses the immune mechanisms that govern physiological tissue healing, as well as the age-related perturbations that lead to ulcerative and fibrotic diseases. It also highlights the potential application of extracellular vesicles (EVs), both mammalian and plant-derived, as a stable and low-immunogenicity mediator to modulate and re-establish repair homeostasis. Translational hurdles such as EV standardization, dosing, safety assessment, and manufacturing are critically discussed to promote their use in geroscience, regenerative medicine, and dermatology.

## 1. Introduction

Tissue repair is a finely coordinated, multistep biological process that restores tissue integrity following injury through the sequential activation of hemostatic, inflammatory, proliferative, and remodeling phases [1]. Each stage is tightly regulated in time and space by the interplay between resident and recruited cells, soluble mediators, extracellular matrix (ECM) components, and mechanical cues, ensuring effective healing and the resolution of inflammation.

Aging profoundly impairs the efficiency of tissue repair. With advancing age, alterations in immune function, stem and progenitor cell activity, cellular senescence, and ECM remodeling collectively delay healing and promote chronic inflammation and fibrosis [2]. These changes result in a diminished capacity to restore tissue homeostasis, increasing vulnerability to non-healing wounds and maladaptive repair responses. Defective tissue repair represents a major clinical burden in aging populations and underlies the pathogenesis of several age-related diseases [3]. Chronic wounds, including venous leg ulcers, diabetic foot ulcers, and pressure injuries, are highly prevalent in older adults and are characterized by persistent inflammation, impaired angiogenesis, and failure of the re-epithelialization process [4,5]. Similarly, fibrotic disorders and vascular diseases emerge from an unresolved or dysregulated repair, leading to excessive ECM deposition, tissue stiffening, and progressive loss of organ function. Together, these conditions impose substantial morbidity, mortality, and socioeconomic costs on healthcare systems worldwide.

In recent years, extracellular vesicles (EVs) have emerged as critical mediators of intercellular communication, as well as in tissue repair and immune regulation. EVs transport a diverse cargo of proteins, lipids, nucleic acids, and metabolites, enabling them to modulate inflammation, angiogenesis, cell proliferation, and matrix remodeling [6,7,8]. Among the different classes of EVs, plant-derived EVs (PDEVs) and mammalian-derived EVs (MDEVs) have gained increasing attention as innovative therapeutic candidates. These vesicles exhibit high stability, low immunogenicity, and intrinsic biocompatibility and can be cost-effectively produced at scale. Moreover, growing evidence indicates that these models can modulate immune responses, oxidative stress, and tissue regeneration through cross-kingdom signaling mechanisms, making them particularly attractive for the treatment of chronic and age-associated repair disorders [6,8].

Aging-associated dysregulation of tissue repair represents a major unmet clinical need, and the identification of new promising strategies to restore repair homeostasis and immune balance in age-related diseases is an emerging research tool.

## 2. Orchestrating Cutaneous Wound Regeneration, Repair, and Healing: The Pivotal Role of Innate Immune Cells

Healing refers to the entire process by which the organism responds to tissue injury, such as wounds, through regeneration and repair programs to ensure functional integrity. The outcome depends on damage severity, tissue type, host systemic conditions, and, crucially, the tissue’s ability to activate regeneration during inflammation resolution [9]. This process may result in the complete restoration (*restitutio ad integrum*) of the damaged tissue, often aligning with true tissue regeneration, or in the scar formation through reparative substitution.

Regeneration is a fundamental biological process of healing by which cells, tissues, organs and even entire limbs are restored after injury or disease. It can occur in spectacular ways, as some organisms are capable of completely regenerating themselves from a small group of cells [10]. A well-known example is the liver: following partial damage or surgical resection, the remaining hepatic tissue expands to restore organ mass and functional capacity. Comparable adaptive responses have been observed in other organs, such as the pancreas, thyroid, kidneys, adrenal glands, and lungs, although their regenerative potential is more restricted [11]. The basis of regenerative potential is the presence of stem cells, programmed to differentiate into the tissue cells that they will replace. Numerous studies have been conducted on the mechanisms that enable stem cells to orient themselves and subsequently differentiate into a specific cell type. These studies have also investigated the potential for reversing this process, thereby transforming adult cells into stem cells or restoring the capacity of stem cells to proliferate and differentiate. This capacity is often lost during the aging process, concomitant with the depletion of the stem cell pool and the impairment of their quiescence and self-renewal [12].

Unlike regeneration, repair can result in unresolved inflammation, fibrosis, and scaring [13]. The repair process prevails when regeneration is overwhelmed by persistent injury or when regenerative capacity is inherently limited due to the type of cells that make up the damaged tissue or the extent of the injury [14,15].

**Figure 1:**
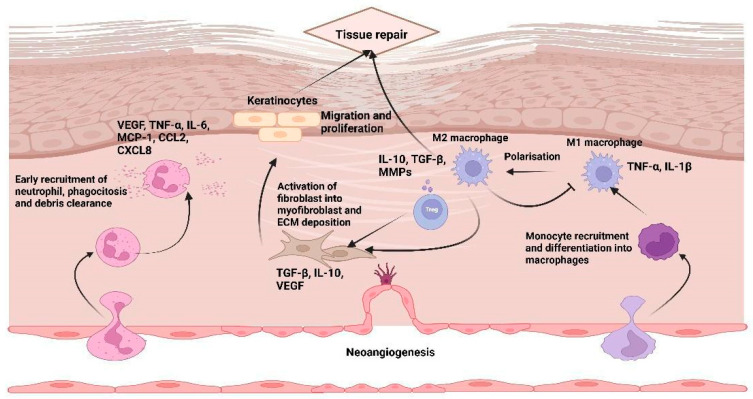
Schematic representation of acute, self-limiting cutaneous wound healing following tissue injury. The inflammatory phase is initiated by early neutrophil recruitment and debris clearance, followed by macrophage polarization from a pro-inflammatory M1 phenotype (TNF-α, IL-1β) to a reparative M2 phenotype (IL-10, TGF-β). Tregs contribute to inflammation resolution. During the proliferative phase, keratinocytes migrate and proliferate, activate fibroblasts and myofibroblasts deposit-controlled ECM, and endothelial progenitor cells promote VEGF-mediated angiogenesis and granulation tissue formation. The process culminates in immune cell clearance and ECM remodeling, restoring tissue integrity. Created by BioRender. Calabrò, A. (2026) https://BioRender.com/xiudnmb (accessed on 27 February 2026).

A perfect model of the repair process is provided by cutaneous wound healing (Figure 1). Wounds arise from surgery, trauma, extrinsic factors (pressure, burns, cuts), or pathologies like diabetes and vascular disorders. In general, wound healing efficiency reflects the interplay of local pathology and host-environment factors. Advanced age, vascular/metabolic/immune diseases, and pharmacological treatments profoundly influence the regeneration and repair trajectories. An ideally healed wound is defined as one that fully restores normal tissue structure, function, and anatomical appearance following injury. A minimally healed wound, by contrast, is characterized by the re-establishment of anatomical continuity without sustained functional recovery, rendering the tissue susceptible to recurrence. Between these extremes, an adequately healed wound achieves stable restoration of both anatomical and functional continuity.

Based on their etiology and clinical course, wounds are commonly classified as acute or chronic. Acute wounds generally progress through a well-coordinated repair process, ultimately leading to the stable restoration of anatomical integrity and tissue function. In contrast, chronic wounds fail to complete the healing process effectively and remain trapped in a state of incomplete repair, thereby preventing full anatomical and functional recovery [16].

In the human skin, the phase that immediately follows tissue injury is the inflammatory one, which encompasses both the hemostatic process and the inflammatory response properly, with the active involvement of cells of the immune system. Initially, hemostasis is activated, consisting of the coagulation cascade [17] and the formation of platelet clots [16]. Platelets adhere to exposed subendothelial structures (i.e., collagen, fibronectin) via receptors (e.g., glycoprotein (GP)Ib, GPIc/IIa) with von Willebrand factor as a mediator. Platelet aggregation with fibrinogen forms a provisional clot that entraps erythrocytes and leukocytes, creating a red thrombus [17]. This clot not only prevents bleeding but also provides a scaffold for the migration of epidermal cells, endothelial cells, fibroblasts, and immune cells, thereby preparing the wound bed for subsequent healing phases. Activated platelets form a fibrin-rich clot and release a wide array of cytokines, growth factors and platelet-derived EVs (PEVs) enriched in pro-coagulant molecules and chemokines [18]. These mediators stabilize the clot, recruit neutrophils and monocytes, and prime angiogenesis, effectively bridging hemostasis with the onset of inflammation and establishing the microenvironment required for tissue renewal. Furthermore, beyond their hemostatic role, platelets act as central hubs of inflammation, coordinating immune–vascular crosstalk through the release of EVs and surface interactions with leukocytes and endothelial cells [19]. In addition, there is the recognition of the injurious agent and the activation and recruitment of innate immune cells, such as neutrophils, mast cells, monocytes, and macrophages [9,16]. A major role in this process is that of reactive oxygen species (ROS), primarily produced by platelets, which contribute to clot formation. At low concentrations, they act as signaling molecules that promote the recruitment of immune cells involved in the initial inflammatory response. As the process progresses, higher ROS levels exert potent antimicrobial effects, supporting host defense against invading pathogens [20].

Within hours of injury, neutrophils infiltrate the wound to clear microbes and debris via phagocytosis, ROS production and neutrophil extracellular traps (NETs); these cells also shape vascular permeability and recruit monocytes [21]. While both NETs and ROS contribute to antimicrobial defense, an excess of their production impairs healing and sustains inflammation [15,20]. Notably, aging alters neutrophil function and heterogeneity, predisposing to prolonged inflammation and delayed repair [22]. Following this, as inflammation progresses, circulating monocytes infiltrate the wound and differentiate into macrophages that orchestrate both the persistence and resolution of inflammation. In the early phase, pro-inflammatory M1 macrophages predominate, releasing tumor necrosis factor (TNF)-α, interleukin (IL)-1β and nitric oxide (NO) to support pathogen clearance and T helper cell (Th)1 activation. Subsequent efferocytosis of apoptotic neutrophils and shifts in cytokine and lipid mediators trigger their conversion to anti-inflammatory, pro-reparative M2 macrophages. These cells secrete IL-10, transforming growth factor (TGF)-β, and vascular endothelial growth factor (VEGF), recruit fibroblasts and endothelial cells, and drive the transition from the inflammatory to proliferative phase [23]. M2 macrophages perform dual roles: clearing apoptotic neutrophils and cellular debris (efferocytosis) and releasing pro-resolving mediators that suppress further inflammation while promoting tissue repair [4]. Indeed, macrophages release factors that favor the migration of cells involved in the proliferative phase and differentiating into M2/M3 type, participating in the re-epetilization phase [16].

In the proliferative and remodeling phases of healing in the cutaneous model, additional immune and stromal cell types intervene. T lymphocytes, particularly regulatory T cells (Tregs) and certain effector subsets, guide the recruitment and activation of fibroblasts and endothelial cells [17].

Fibroblasts are recruited to the wound bed from the circulation in response to cytokine stimulation (e.g., IL-4, IL-13 and interferon (IFN)-γ), then proliferating and producing ECM components (such as glycosaminoglycans and proteoglycans), taking the name of myofibroblasts, giving rise to granulation tissue, a temporary but essential scaffold for repair [16,24]. The defining feature of these cells is their contractile ability, which drives the formation of granulation tissue.

Growth factors and cytokines, including TGF-β, VEGF, and platelet-derived growth factor (PDGF), stimulate angiogenesis, ECM deposition, and the differentiation of fibroblasts into myofibroblasts. These processes are essential for wound contraction and scar formation, which typically begin after the second week of injury. Meanwhile, surviving parenchymal cells or resident stem/progenitor cells proliferate to replace lost functional tissue wherever regeneration is possible [24].

The process of re-epithelialization, which represents an example of a regeneration process, begins during this phase and is characterized by the proliferation and migration of keratinocytes from the wound margins toward the central area of the lesion, occurring alongside the formation of granulation tissue that fills the space between the wound bed and edges. Overall, cutaneous re-epithelialization reflects a coordinated reorganization of keratinocytes, allowing the restoration of the basal layer and the subsequent recovery of the stratified structure of the epidermis through proliferation and differentiation. They promote the re-epithelialization phase through the production of growth factors (fibroblast growth factor (FGF), keratinocyte growth factor (KGF)), while in the early reparation stages, keratinocytes can produce cytokines that support the activation and functionality of fibroblasts, creating a paracrine loop [25,26,27]. This crosstalk is crucial for terminating the reparative phase and preventing the development of hypertrophic or fibrotic scars.

The remodeling phase typically begins approximately three weeks after the injury and may extend for over a year. During this phase, processes activated in earlier stages are progressively attenuated, and transient repair-associated cells, including macrophages, endothelial cells, and myofibroblasts, undergo apoptosis or are cleared from the wound site. This results in tissue being predominantly composed of collagen and other ECM components.

Dynamic interactions between the epidermis and dermis, supported by feedback mechanisms, allow long-term regulation of skin integrity and tissue homeostasis. Within this context, type III collagen initially deposited in the ECM is gradually replaced by type I collagen over a period of 6–12 months, contributing to the mechanical strengthening of the repaired tissue. Keratinocytes play an important role in this framework. They produce stratifin (14-3-3σ), which acts to induce fibroblasts to release matrix metalloproteases (MMPs), which degrade excess ECM components and simultaneously inhibit the deposition of collagen types I and III [25,26,27]. Interestingly, stratifin expression is in turn regulated by fibroblasts themselves via the secretion of growth factors such as KGF/FGF7, IL-1α, granulocyte colony-stimulating factor, and pannexin-3, which act as key mediators of the dynamic crosstalk between keratinocytes and fibroblasts [26].

Ultimately, the balance between regeneration and fibrosis determines the quality of the healed tissue. A well-coordinated resolution process restores normal structure and function; a dysregulated or incomplete process may culminate in chronic inflammation, or pathological scarring.

Although the underlying sequence of events (hemostasis, inflammation, proliferation and re-epithelialization), described for the cutaneous district, is broadly conserved across different tissues, the process is generally dynamic, multifactorial, and influenced by external and internal modulators, like aging itself [24].

## 3. Dysregulated Tissue Repair in Aging: Immunosenescence as One Culprit

Impaired repair mechanisms in older people involve stem cell dysfunction, diminished angiogenesis, chronic low-grade inflammation (inflammaging), and immune system dysfunction (immunosenescence) [24,28,29]. These conditions lead to increased healing times, chronic inflammation, aberrant tissue repair, fibrosis, organ dysfunction, and tumorigenesis, mediated mainly by cellular senescence in critical cells for healing [30]. In this context, the metabolic product of senescent cells, the senescence-associated secretory phenotype (SASP), has highly context-dependent effects. In physiological processes, SASP components, including granulocyte–macrophage colony-stimulating factor and chemokines like CXCL1, play a beneficial role by attracting immune cells, such as macrophages and neutrophils, to sites of injury. This coordinated recruitment facilitates the clearance of senescent cells, supports tissue regeneration, and contributes to the restoration of normal tissue structure [31].

In contrast, with the aging and chronic pathologies, sustained SASP activity becomes detrimental. Persistent secretion of pro-inflammatory mediators promotes chronic inflammation, ECM remodeling, and tumorigenesis. For instance, factors such as TNF-α and MMPs are known to amplify inflammatory responses and drive matrix degradation, thereby worsening tissue damage and dysfunction [31].

The accumulation of senescent cells and SASP also leads to excessive ROS production, resulting in oxidative damage to key macromolecules involved in tissue repair. This imbalance triggers the activation of nuclear factor kappa-light-chain-enhancer of activated B cells (NF-κB), which amplifies inflammatory signaling and promotes the expression of pro-inflammatory cytokines such as IL-1, IL-8, and TNF-α, thereby establishing a self-perpetuating inflammatory loop. Hydrogen peroxide induces the expression of P-selectin on endothelial cells, enhancing leukocyte adhesion and prolonging inflammation [20].

In chronic wounds, M1 macrophages also contribute to the inflammatory environment by the release of high levels of TNF-α and hydroxyl radicals, which further activate the NLRP3 inflammasome, leading to increased secretion of IL-1β [32]. This cytokine reinforces the inflammatory response and inhibits the transition toward the anti-inflammatory M2 phenotype, thereby impairing resolution of inflammation and delaying healing.

Excessive ROS production also disrupts key processes required for tissue regeneration, including angiogenesis and ECM remodeling. In particular, ROS interfere with angiogenesis by inhibiting prolyl hydroxylase domain proteins, resulting in the abnormal stabilization of hypoxia-inducible factor-1α even under normoxic conditions. This dysregulation leads to the formation of immature and leaky blood vessels, ultimately compromising effective tissue repair. Moreover, oxidative stress impairs keratinocyte proliferation and migration, which are essential for re-epithelialization [31].

In the later stages of wound healing, persistent oxidative stress predisposes tissues to fibrotic and aberrant proliferative responses. This is largely mediated by the overactivation of TGF-β, which promotes the differentiation of fibroblasts into myofibroblasts. These cells produce excessive amounts of collagen, fibronectin, and other ECM components, contributing to pathological fibrosis. In addition, TGF-β inhibits myofibroblast apoptosis and disrupts ECM turnover by suppressing MMP activity while increasing the expression of tissue inhibitors of metalloproteinases, thereby favoring matrix accumulation. Oxidative stress also activates the p38 MAPK signaling pathway, enhancing lysyl oxidase activity and promoting abnormal collagen cross-linking. Collectively, these mechanisms contribute to the development of hypertrophic scars and keloids.

Another impaired mechanism that contributes to impaired wound healing is supported by the alteration of the mammalian target of rapamycin (mTOR)-AMPK pathway. In particular, since many aging-related stresses (oxidative stress, inflammatory mediators, and ECM stiffness) drive myofibroblast formation, AMPK signaling serves as a countermeasure that reduces fibrosis, slows degenerative remodeling of the ECM and attenuates fibroblast hyperactivation during aging [33]. This is achieved by the inhibition of mTOR complex 1, which contributes to reduced myofibroblast activation. By inhibiting mTOR, AMPK reduces excessive ECM synthesis, SASP expression and inflammaging [33]. Thus, mTOR promotes fibrogenesis via upregulation of collagen synthesis, supporting myofibroblast contractility and inhibiting autophagy (thereby accelerating senescence). Together, AMPK and mTOR form an antagonistic pair controlling metabolic homeostasis. In aging tissues, a shift toward chronic mTOR activation and AMPK suppression contributes to cellular senescence, inflammaging, ECM dysregulation and fibrosis. By contrast, AMPK activation reverses many of these changes.

Moreover, hormonal/genetic components, including estrogenic or androgenic modulation of inflammation, epidermal function, and ECM genes, contribute to these impairments, ultimately predisposing aging individuals to specific pathologies where dysregulated tissue repair mechanisms predominate.

### 3.1. Ulcerations: A Paradigm of Immune Dysregulation in Aging

As aging progresses, the skin undergoes profound structural and functional alterations that impair wound healing and increase susceptibility to chronic, non-healing wounds. While aging is a major risk factor for chronic ulcer development, it rarely acts in isolation. Chronic wounds in older individuals are frequently compounded by age-associated comorbidities, such as cardiovascular disease, diabetes, and cancer, which further disrupt vascular function, immune regulation and, consequently, tissue repair mechanisms.

The incidence of ulceration and cutaneous chronic wounds in the older population over the age of 65 has increased significantly compared to the general population, with, for example, 4–5% of the population affected by venous ulcers [34]. Venous leg ulcers (VLUs) associated with chronic venous insufficiency represent the most common form of chronic wounds, affecting several hundred thousand individuals. These ulcers typically develop on the lower leg, most frequently near the medial malleolus, and arise because of persistent venous hypertension. Clinically, VLUs are characterized by oedema, skin induration, hemosiderin deposition, and stasis dermatitis. Major risk factors include a prior history of deep vein thrombosis, long-standing venous disease, multiple pregnancies, and advancing age.

Arterial ulcers, by contrast, most often occur on the feet and toes and are associated with severe pain, impaired blood flow, and sharply demarcated lesions with a characteristic “punched out” appearance. These wounds frequently display necrotic tissue, particularly in distal regions such as the toes. Chronic ulcers resulting from peripheral arterial disease are a major contributor to wound-related mortality and long-term disability, with a frequency of 85% in the population over 65 years old [34,35]. Notably, a significant proportion of patients with chronic wounds present with mixed arterial and venous pathology, a condition linked to slower healing and higher recurrence rates.

Diabetic foot ulcers (DFUs) may develop through neuropathic, ischemic, or combined mechanisms. DFUs affect approximately 18.6 million people worldwide each year, with higher prevalence in older adults, and have a 5-year mortality rate of roughly 30% [36,37]. Sensory deficits, structural foot deformities, and reduced vascular supply predispose individuals to ulcer formation at pressure-bearing sites, including the plantar surface, malleoli, dorsum of the foot, and distal toes.

In contrast, pressure ulcers result from prolonged, unrelieved pressure over bony prominences, such as the sacrum and heels, and are exacerbated by shear forces, friction, and immobility, conditions commonly encountered in older adults [38].

A subset of atypical wounds can resemble more common ulcer types, complicating diagnosis. These include malignant ulcers, such as squamous cell carcinoma (SCC) arising in chronic wounds, as well as vasculitic lesions associated with systemic inflammatory diseases.

Non-healing ulcers or those exhibiting atypical features, such as irregular or elevated margins or excessive granulation tissue unresponsive to conventional treatment, should prompt further diagnostic evaluation, including biopsy. Other less common causes, such as fungal or mycobacterial infections, pyoderma gangrenosum, and autoimmune disorders, should also be considered, particularly when ulcers present in unusual locations or are accompanied by systemic symptoms [34,35].

**Figure 2:**
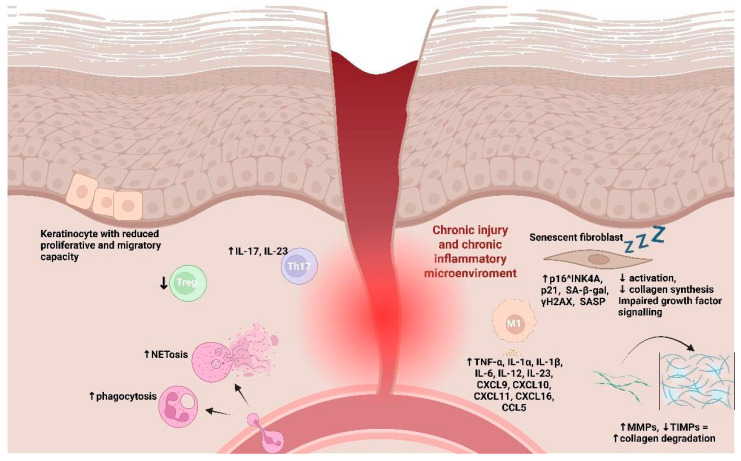
Schematic representation of chronic inflammation as the central driver of impaired cutaneous wound healing. Persistent inflammatory signaling, sustained by continuous production of pro-inflammatory mediators (e.g., IL-6, TNF-α, ROS), promotes prolonged neutrophil infiltration and excessive NETosis, leading to defective resolution of the inflammatory response. Chronic inflammation also induces dysregulated macrophage polarization (M1/M2 imbalance) and the accumulation of senescent immune cells and fibroblasts (p16INK4, p21), further compromising tissue repair. These alterations impair angiogenesis, disrupt fibroblast function, and delay re-epithelialization, resulting in aberrant ECM deposition and progressive fibrosis. The persistent failure to restore immune–stromal homeostasis ultimately culminates in chronic wounds, fibrotic remodeling, and tissue degeneration. Created by BioRender. Calabrò, A. (2026) https://BioRender.com/xiudnmb (accessed on 27 February 2026).

From a pathological point of view, the progression of chronic wounds and ulcers in aging arises from the disruption of the tightly regulated mechanisms that govern tissue repair. A defining feature of age-related impaired healing is the failure to properly resolve the inflammatory phase, which prolongs tissue damage and delays progression to subsequent healing stages (Figure 2) [39].

In older individuals, the inflammatory response is often skewed toward a pro-inflammatory state, characterized by excessive neutrophil infiltration and persistent production of cytokines, growth factors, and other soluble mediators. Elevated neutrophil-to-lymphocyte ratios, enhanced NET formation, and delayed clearance of inflammatory signals collectively impair the resolution of inflammation, a phenomenon further exacerbated by immunosenescence and low-grade chronic inflammation.

Age-related wounds and, particularly, diabetic ones, whose incidence increases with the aging process, also exhibit an imbalance in macrophage polarization, with a predominance of pro-inflammatory M1 macrophages over reparative M2 macrophages, accompanied by reduced secretion of anti-inflammatory cytokines (i.e., IL-10 and TGF-β) and growth factors essential for the transition to the proliferative phase.

Altered monocyte and macrophage function in chronic wounds further impacts adaptive immunity, leading to reduced Treg cell activity and increased infiltration of Th17 cells, which amplifies neutrophilic inflammation and prolongs the inflammatory phase. Thus, the proliferative phase, normally driven by fibroblasts, keratinocytes, mast cells, and M2 macrophages, is profoundly compromised. Indeed, during the proliferative phase, in aged skin models, keratinocytes fail to respond adequately to critical growth factors, such as FGF or epidermal growth factor, which limit re-epithelialization. With aging, these cells undergo morphological changes, including alterations in size and shape, which impair their migratory and proliferative capacity [27]. Their function is further compromised by impaired crosstalk with mesenchymal cells and immune cells, which normally secrete cytokines and other mediators essential for keratinocyte proliferation, migration, and differentiation [27]. Furthermore, experiments conducted *in vivo* on mouse models suggested that another aging-related alteration could impair the functionality of keratinocytes. It has been shown that the depletion of autophagy-related 5/7 genes in mice inhibited the autophagy capacity of keratinocytes, acting on the production of the transcription of the CCL2 gene, induced by TNF. This reduces the capacity of CCL2 to activate the fibroblast and to recruit immune cells essential for wound repair [40].

Melanocytes are an often-overlooked component of skin aging and play a role in tissue repair mechanisms [41]. Located in the basal layer of the epidermis, melanocytes contribute to fibroblast proliferation. Their activation triggers the production of α-melanocyte-stimulating hormone, which promotes T cell activation and TGF-β production. TGF-β then stimulates fibroblast proliferation via the SMAD3 and SMAD6 signaling pathways, which can also influence the formation of pathological scars under certain conditions. In aged skin, senescent melanocytes have been identified as the predominant cells expressing p16INK4A [42]. These senescent cells contribute to epidermal thinning, flattening of the dermo-epidermal junction, and reduced proliferation of keratinocytes. They also impair fibroblast participation in wound repair, thereby compromising the skin’s regenerative capacity. Thus, melanocytes have a dual role: they support normal tissue repair in young, healthy skin, but their senescence during aging contributes to impaired wound healing and structural deterioration of the skin. In addition, age-associated stem and progenitor cell dysfunction reduces the proliferative capacity of resident skin cells and circulating endothelial progenitors, while chronic oxidative stress, impaired angiogenic signaling, and cumulative cellular damage hinder re-epithelialization. Consequently, neovascularization is diminished, granulation tissue formation is delayed, and wound closure may fail [39].

Effective wound closure also relies on dermal ECM reconstruction, which provides the structural scaffold for epidermal coverage. Fibroblasts and myofibroblasts mediate this process, supported by macrophages, mast cells, and lymphocytes via VEGF and TGF-β-dependent mechanisms. In chronic wounds, however, macrophage polarization remains skewed toward a pro-inflammatory phenotype, while fibroblasts exhibit reduced activation, decreased collagen synthesis, and impaired growth factor signaling, further limiting ECM deposition and tissue regeneration.

The final remodeling phase consolidates granulation tissue into mature scar tissue and partially restores tissue function. Fibroblasts, myofibroblasts, and M2 macrophages regulate controlled collagen turnover, replacing type III collagen with type I collagen under the guidance of growth factors and matrix-modifying enzymes. A balanced interplay between MMPs and their inhibitors ensures proper scar maturation and tensile strength. In contrast, age-impaired wounds are characterized by persistent hypoxia, oxidative stress, defective angiogenesis, and sustained pro-inflammatory macrophage activity, all of which contribute to aberrant ECM remodeling. Age-associated fibroblast dysfunction produces thinner, disorganized matrices, further reducing the regenerative capacity of the wound bed.

Interventions that restore macrophage polarization, enhance fibroblast activity, or supplement ECM components show promise in partially reversing these deficits, highlighting potential strategies to improve repair outcomes in aging populations.

### 3.2. Dysregulated Fibrosis in Aging: “Fibroaging” as a Hallmark

Fibrosis is an evolutionarily conserved repair response that is activated when tissues sustain severe or chronic injury and regenerative potential is exhausted or insufficient for complete restoration. It represents a common pathological endpoint of many chronic diseases, where disease-specific insults trigger tissue damage that is accompanied by persistent inflammation or aberrant activation of resident cells, such as epithelial cells and the microvascular endothelium [27]. So, when the healing process becomes dysregulated, repair often progresses toward a sustained and aberrant fibrotic remodeling program characterized by the overactivation of core profibrotic signaling pathways, including TGF-β, and other growth factors, as well as multiple self-amplifying feedback loops. Despite the diversity of initiating insults, a unifying feature of fibrotic diseases is the activation and persistence of myofibroblasts, which act as the principal effector cells driving fibrotic remodeling. The resulting scarring process is marked by excessive and disorganized deposition of ECM components, predominantly fibrillar collagens, leading to distortion of normal tissue architecture and progressive loss of organ function [43].

Although aberrant fibrotic processes can occur at any age, the incidence of pathological fibrosis increases significantly with aging. Age-related diseases characterized by fibrotic manifestations include idiopathic pulmonary fibrosis, liver fibrosis associated with non-alcoholic fatty liver disease, chronic kidney disease, and cardiac fibrosis linked to cardiovascular events [43]. Selman and Pardo [44], to underline the striking bond between fibrosis and aging, used the term “fibroaging”, which refers to the progressive dysfunction of fibroblasts, particularly dermal fibroblasts, driven by altered interactions with the ECM and mechanical environment, resulting in tissue degeneration and fibrosis. At the basis of this alteration, cellular senescence plays an important role because the accumulation of senescent fibroblasts is a hallmark of aging. These cells enter permanent growth arrest but remain metabolically active, releasing inflammatory cytokines, proteases, and growth factors (SASP) that disrupt tissue homeostasis [44].

The role of senescent fibroblasts, particularly myofibroblasts, is dual. *In vitro* studies have suggested that stress-induced senescence in myofibroblasts may act as a brake on the formation of aberrant scars, such as keloids. Accumulating evidence indicates a genetic predisposition to keloid development, with ethnic background emerging as an important contributing factor. The highest prevalence has been reported in African populations, followed by Asian populations, although the precise genetic determinants underlying this susceptibility remain incompletely understood and continue to be an active area of investigation [45]. It has been hypothesized that keloid fibroblasts undergo senescence at a slower rate than fibroblasts in normal scar tissue, continuing to deposit collagen and other ECM proteins beyond the levels typically observed in physiological wound healing [46]. This defective induction of the senescent phenotype reduces the number of senescent cells, thereby diminishing their inhibitory effect and allowing uncontrolled cellular proliferation, ultimately leading to keloid formation. Key molecular mediators, including CCN1 and TGF-β, are thought to regulate this process by promoting senescence in fibroblasts and limiting excessive ECM deposition. When these regulatory pathways are insufficient, the senescence-associated inhibition is unable to restrain fibroblast proliferation, resulting in hypertrophic or keloid scar development [46].

#### The Emblematic Example of Liver Fibrosis as a Chronic Wound Repair Result

**Figure 3:**
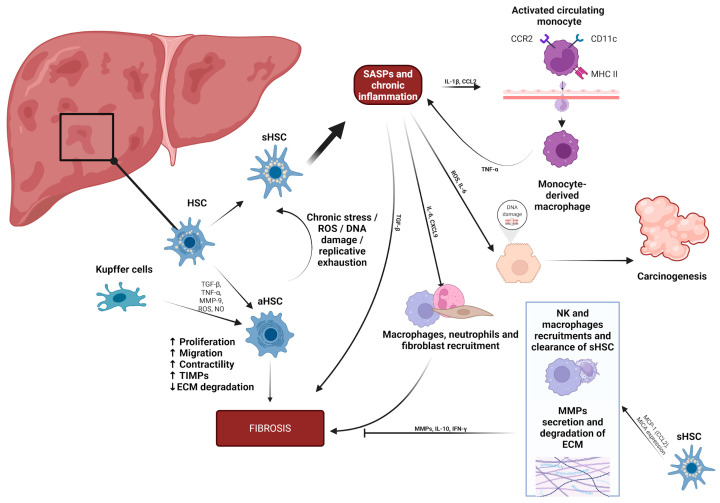
Schematic representation of chronic inflammation-driven liver fibrosis and carcinogenesis. Chronic liver injury promotes hepatocyte damage, oxidative stress (ROS), DNA damage, and replicative exhaustion, leading to activation of HSCs into activated HSCs. Kupffer cells contribute to this process by releasing pro-fibrogenic and pro-inflammatory mediators (e.g., TGF-β, TNF-α, MMPs, ROS, NO), further enhancing HSC activation. Activated stellate cells acquire proliferative, migratory, contractile, and ECM-producing properties, driving excessive ECM deposition and fibrosis. SASPs and chronic inflammation sustain recruitment of circulating immune cells and monocyte-derived macrophages through cytokines and chemokines (e.g., IL-1β, CCL2, TNF-α). Persistent immune cell infiltration, together with macrophage-, neutrophil-, and fibroblast-mediated signaling, amplifies inflammatory and fibrogenic pathways. Although NK cells and macrophages can mediate clearance of senescent HSCs and promote ECM degradation via MMPs, IL-10, and IFN-γ, failure to restore immune–stromal homeostasis favors progressive fibrosis. Sustained inflammation and genomic instability ultimately contribute to hepatocarcinogenesis [52]. Created by BioRender. Calabrò, A. (2026) https://BioRender.com/xiudnmb (accessed on 27 February 2026).

A complex example of age-related disease connected to fibroaging is the hepatic one (Figure 3). Liver fibrosis is a common feature of chronic liver diseases, including non-alcoholic fatty liver disease, and is characterized by the excessive deposition of ECM components. It is an intermediate stage that can progress to cirrhosis and end-stage liver failure. These conditions are responsible for over one million deaths worldwide each year [47].

The defining feature of liver fibrosis is the persistent activation of fibrogenic myofibroblasts, which drives the accumulation of abnormal ECM and the distortion of the hepatic tissue’s architecture. This activation is sustained by a complex network of paracrine signals, including PDGF and TGF-β, as well as a variety of cytokines and chemokines. These mediators activate intracellular signaling pathways that promote the proliferation, migration and contractility of cells, as well as the synthesis of ECM. This ultimately induces the trans-differentiation of mesenchymal precursor cells, such as pericytes and resident fibroblasts, into activated myofibroblasts, while maintaining their fibrogenic phenotype [47]. In this context, TGF-β plays a predominant role, and several mechanisms are being studied to block its abnormal action during fibrosis. TGF-β signals by binding to the TGF-β type II receptor (TβR2) on the cell membrane, which then recruits and phosphorylates the type I receptor (TβR1). This subsequently leads to the phosphorylation and activation of the SMAD2 and SMAD3 transcription factors, which promote the expression of various fibrosis-related genes. TGF-β also stimulates SMAD-independent, non-canonical pathways (i.e., mitogen-activated protein kinases), which further amplify profibrotic responses. KLOTHO, a protein known as an anti-aging factor, seems to act as an anti-fibrotic agent [48]. A KLOTHO-derived peptide, acting as an antagonist of TβR2, suppressed both canonical and non-canonical TGF-β signaling in hepatic stellate cells (HSCs) in murine models of aging and spontaneous hepatic fibrosis. Infusion of this protein restored hepatic integrity and function in mice, mitigating fibrosis.

HSCs constitute the principal source of myofibroblasts in the injured liver [49]. In physiological conditions, HSCs remain quiescent and express characteristic markers, including PDGF receptor β and desmin. Upon chronic liver injury, these cells acquire a myofibroblast-like phenotype, gaining the capacity to secrete ECM proteins and pro-inflammatory mediators. Resolution of fibrosis normally involves apoptosis, inactivation, or senescence of activated HSCs once the fibrogenic stimulus subsides. However, while cellular senescence may limit fibrosis, it can also impair regenerative capacity and, in certain contexts, contribute to a pro-tumorigenic microenvironment, particularly when senescence spreads to adjacent hepatocytes [49].

The immune system plays a pivotal role in regulating both the progression and resolution of liver fibrosis (Figure 3). Among immune cells, macrophages represent a highly heterogeneous population, accounting for approximately 10–15% of total liver cells, and exert both pro- and anti-fibrogenic effects through paracrine regulation of HSC activation [50]. Experimental depletion of macrophages in murine models has revealed their essential contribution to fibrogenesis. In models of fibrotic livers, it has been demonstrated that Ly6C^high^ monocyte-derived macrophages are recruited in a CCR2-dependent manner and secrete a broad array of pro-fibrogenic mediators, including TGF-β, PDGF, and multiple chemokines that amplify inflammation and HSC activation. In contrast, Ly6C^low^ macrophages represent a fibrolytic subset that expands during fibrosis regression [50]. Their differentiation is driven by CX3CR1 signaling, and these cells promote apoptosis of activated HSCs while facilitating ECM degradation through secretion of matrix metalloproteinases such as MMP-12 and MMP-13. Galectin-3, a macrophage-derived lectin, has emerged as a key pro-fibrotic mediator that enhances HSC activation and is currently being targeted pharmacologically in clinical trials for non-alcoholic steatohepatitis.

Adaptive immune cells also modulate fibrogenesis. Th17 cells, which secrete IL-17A and IL-22, are increased in both liver tissue and circulation in chronic liver diseases. IL-17 signaling in macrophages and HSCs induces pro-inflammatory cytokine production and collagen type I expression via STAT3 activation, thereby exacerbating fibrosis. Conversely, IL-22 has context-dependent effects: while it can attenuate fibrosis by inducing HSC senescence through STAT3- and p53-dependent mechanisms, it may exert detrimental effects in specific settings such as viral hepatitis [51].

Tregs also influence fibrogenesis by modulating innate immune responses. In chronic hepatitis, intrahepatic Foxp3^+^CD4^+^ Tregs producing IL-8 have been shown to activate HSCs and promote fibrosis. In contrast, γδ T cells exert antifibrotic effects by inducing HSC apoptosis in a CCR6-dependent manner [51].

B cells, which can constitute up to half of hepatic lymphocytes, also contribute to fibrogenesis. Innate B cell activation mediated by HSCs through MyD88-dependent pathways promotes liver fibrosis in experimental models, highlighting an additional layer of immune–stromal interaction [51].

Natural killer (NK) and NK T (NKT) cells further shape fibrotic outcomes. NK cells display potent antifibrotic activity by selectively killing senescent and early activated HSCs and by secreting IFN-γ, which induces HSC apoptosis and cell cycle arrest. This activity is enhanced by IL-15 but can be suppressed by regulatory CD4^+^ T cells, thereby favoring HSC survival. NKT cells exhibit functional heterogeneity: while some subsets exert antifibrotic effects through IFN-γ production and cytotoxicity, others promote fibrosis by secreting IL-4, IL-13, osteopontin, and hedgehog ligands, particularly via the CXCR6-CXCL16 axis in chronic liver diseases such as nonalcoholic steatohepatitis [51].

Given the complexity of tissue repair processes and the central role of fibrosis in chronic liver injury, the development of therapeutic strategies capable of selectively targeting the cellular populations driving pathological remodeling represents a major clinical objective. In this context, an optimal therapeutic approach should finely modulate cellular senescence, preserving its protective and antifibrotic functions while limiting its detrimental, pro-degenerative effects. EVs emerge as a promising tool to achieve this balance, owing to their ability to deliver bioactive cargo in a cell-specific and context-dependent manner, thereby enabling precise regulation of fibrogenic and immune responses.

### 3.3. Dysregulated Repair–Fibrosis Transition in Aging: The Wound Healing–Fibrosis–Cancer (WHFC) Triad

A close mechanistic relationship also exists between wound healing, chronic fibrosis, and cancer risk progression, collectively referred to as the wound healing–fibrosis–cancer (WHFC) triad. These processes represent interconnected outcomes of dysregulated tissue repair and share common molecular and cellular pathways that become increasingly compromised with aging [43].

Myofibroblasts also play a crucial role in this network of processes. In physiological wound healing, myofibroblasts are transiently activated and subsequently cleared during the resolution phase. In contrast, during aging and in age-related fibrotic diseases and cancer, myofibroblasts remain chronically activated, leading to the formation of a dense desmoplastic stroma that favors tissue stiffening, tumor invasion, and therapeutic resistance [43]. Furthermore, the cellular composition of wound granulation tissue closely resembles that of the tumor stroma and includes fibroblasts or cancer-associated fibroblasts, macrophages or tumor-associated macrophages, and endothelial cells. In both physiological wound repair and tumor progression, these stromal components engage in dynamic crosstalk with epithelial or malignant cells through the deposition and remodeling of the ECM and the paracrine release of cytokines and growth factors, including IL-6 and related mediators [43]. In physiological wound healing, this bidirectional communication is tightly regulated and is terminated once re-epithelialization is complete, allowing stromal activation to resolve and tissue homeostasis to be restored. In contrast, in cancer, the persistent and uncontrolled proliferation of malignant cells perpetuates stromal activation, leading to continuous ECM remodeling, angiogenesis, and immune modulation that collectively support tumor growth and invasion. This pathological overlap is particularly evident in melanoma, where lesions often exhibit features of a “non-healing wound” [53], but also in other chronic fibrotic conditions, such as liver cirrhosis or idiopathic pulmonary fibrosis, which are well-recognized precursors to cancer and are strongly associated with aging. Both fibrosis and tumorigenesis arise from a sustained and maladaptive healing response characterized by excessive ECM deposition, persistent stromal activation, and failure to restore tissue homeostasis [43,54]. Indeed, tumors arising in a fibrotic tissue context are more frequent in squamous histology, display accelerated growth kinetics, and tend to localize to peripheral regions [55,56]. Notably, incident pulmonary nodules developing within fibrotic lungs are malignant in approximately 53% of cases, compared with 12.5% in non-fibrotic lungs [57]. Fibrotic lung tissue is characterized by an abundance of fibroblast foci and alternatively activated (M2-like) macrophages, cell populations known to support tumor growth, invasion, angiogenesis, and metastatic dissemination.

Importantly, aberrant fibrosis not only increases the overall risk of cancer development but also predisposes to the emergence of tumors with intrinsically more aggressive and metastasis-prone phenotypes. In this regard, pulmonary fibrosis represents a paradigmatic clinical model of how a chronically unresolved tissue repair program can evolve into a pro-tumorigenic and pro-metastatic microenvironment. These clinical observations provide epidemiological support for molecular frameworks proposing cancer as a pathological extension of dysregulated wound healing. Regarding the therapies, many studies demonstrated that the use of inhibitors of the fibrotic process induced the regression of tumor progression, for example, in the case of lung cancers associated with fibrosis, the use of anti-fibrotic drugs, like inhibitors of TGF-β1 (e.g., pirfenidone) [57].

#### 3.3.1. Marjolin’s Ulcer as Example of WHFC

**Figure 4:**
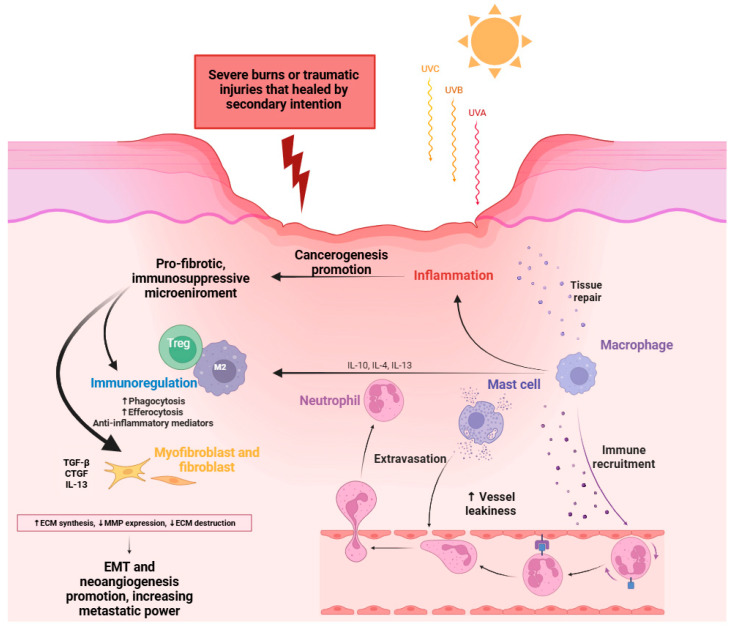
Pathogenic mechanisms underlying MU and inflammation-driven skin carcinogenesis. Persistent inflammation, sustained by UV exposure (UVA, UVB, UVC) and repeated tissue damage, promotes a pro-fibrotic and immunosuppressive microenvironment. Neutrophils, mast cells, and macrophages infiltrate the lesion through enhanced vascular permeability and extravasation, releasing inflammatory mediators and cytokines (e.g., IL-10, IL-4, IL-13) that contribute to immunoregulation and tumor-promoting inflammation. Macrophage polarization toward an M2-like phenotype and Treg-mediated immunosuppression further reinforce immune evasion. Activated fibroblasts and myofibroblasts, stimulated by TGF-β, and IL-13, increase ECM synthesis, matrix remodeling (MMP expression), and tissue stiffening. These processes promote EMT, neoangiogenesis, and progressive acquisition of metastatic potential. Chronic inflammatory signaling and defective tissue repair ultimately favor carcinogenesis, leading to the development of cutaneous malignancies such as squamous cell carcinoma, basal cell carcinoma, and superficial spreading melanoma. Created by BioRender. Calabrò, A. (2026) https://BioRender.com/xiudnmb (accessed on 27 February 2026).

A notable and clinically relevant example of malignant transformation arising from impaired wound repair is Marjolin’s ulcer (MU) (Figure 4). These lesions develop from long-standing, non-healing wounds. They are most associated with non-melanoma skin cancers, such as SCC and basal cell carcinoma (BCC), although melanoma has also been reported [58].

The mechanisms underlying malignant conversion are multifactorial and include persistent inflammatory stimulation, repeated cycles of tissue damage and repair, genetic susceptibility, and exposure to toxic or environmental insults [53].

More broadly, malignancies that arise in areas of defective healing are often referred to as scar tissue-associated cancers. In these settings, the continuous attempts at tissue regeneration are accompanied by sustained cellular proliferation, which increases the likelihood of accumulating genetic alterations over time [59]. In addition, scar tissue is characterized by altered vascularization and impaired immune surveillance, creating a permissive microenvironment in which transformed cells, such as BCC cells, can evade immune detection and expand within a fibrotic niche. Reduced lymphocyte infiltration and disruption of local immune monitoring further contribute to this vulnerability. Clinical observations support this concept. Cases have been described in which BCC developed within scars formed after severe burns or traumatic injuries that healed by secondary intention [60]. These scarred areas, often located in sun-exposed regions, appear particularly susceptible to chronic irritation and ultraviolet-induced damage. Healing by secondary intention emerges as a key risk factor for scar-associated carcinogenesis, as the prolonged loss of epithelial integrity leads to reduced vascular supply, diminished immune defense, and altered collagen architecture. Within this fragile and chronically inflamed environment, phototoxic stress can induce DNA damage and mutations in tumor suppressor genes, ultimately promoting malignant transformation.

#### 3.3.2. Melanoma as Example of Non-Healing Wound-Derived Cancer

Melanomas derived from burn scars are relatively rare, and consequently, MU derived from them. There are only 4 cases reported up to 2018 of multiple melanoma lesions derived from these scars, and a total of only 36 reported cases [61]. In melanoma, defective resolution of immune-mediated tissue repair leads to the persistence of a chronic, wound-like inflammatory microenvironment characterized by sustained activation of pathways normally involved in tissue regeneration [62]. Tumor cell–intrinsic genomic instability promotes micronuclei formation and chronic activation of the cGAS–STING axis, resulting in persistent NF-κB signaling while concomitantly suppressing IFN-I responses. These uncouplings favor pro-metastatic inflammation, epithelial–mesenchymal transition (EMT), and immune evasion rather than effective antitumor immunity. In parallel, melanoma cells hijack regenerative programs through sustained activation of TGF-β, WNT/β-catenin, and NF-κB signaling, acquiring stem-like phenotypes marked by SOX2 and TM4SF1 expression and exhibiting high phenotypic plasticity [62]. The resulting imbalance between inflammatory activation and immune resolution promotes recruitment of immunosuppressive cell populations, including M2-like macrophages and Treg, while impairing cytotoxic T lymphocyte and NK cell surveillance through modulation of major histocompatibility complex class I expression, NK-cell ligands, and immune checkpoints such as programmed death ligand 1. Collectively, these features prevent completion of the remodeling phase typical of physiological wound healing, rendering melanoma metastasis a pathological analogue of a “non-healing wound” that continuously fuels tumor progression and dissemination.

Melanoma-associated stromal cells maintain a chronic inflammatory and pro-angiogenic microenvironment that prevents normal wound resolution. Instead of progressing toward repair, the tissue remains locked in a persistent regenerative-like state that fuels tumor expansion and local invasion. Impaired re-epithelialization and sustained stromal activation contribute not only to tumor progression and dissemination but also to systemic metabolic alterations and cachexia observed in advanced disease. Thus, melanoma and other solid tumors can be viewed as examples of dysregulated tissue repair, in which mechanisms normally devoted to healing are hijacked to sustain malignant growth, highlighting the close conceptual and biological link between chronic wounds and cancer [16].

## 4. Investigating PDEVs and MDEVs as Emerging Strategy to Restore Age-Related Dysfunctions in Tissue Repair

The tissue repair dysfunctions linked to aging have profound implications for public healthcare systems. This underscores the urgent need to enhance repair efficiency in the aging population while minimizing iatrogenic risks in younger individuals. While a wide array of pharmacological, lifestyle-based, and biologic anti-aging strategies is being explored, EV-based interventions may represent one of the most promising mechanistically driven avenues to restore impaired repair processes in aging, complementing rather than replacing these existing approaches [63,64,65].

EVs are membrane-bound nanostructures released by cells across all kingdoms of life and serve as conserved mediators of intercellular communication. By transferring proteins, lipids, and various RNA species, they regulate key physiological processes, including development, immune responses, and tissue repair, while also participating in pathological conditions such as cancer and chronic inflammation.

PDEV biogenesis is a tightly regulated intracellular process that proceeds through multiple pathways. As described by Sun et al. (2025) [66], three principal routes have been identified: (i) the multivesicular body pathway, involving the maturation of early endosomes into late endosomes containing intraluminal vesicles via ESCRT machinery and associated with plant tetraspanins such as TET8 and TET9; (ii) exocyst-positive organelle-mediated secretion; and (iii) vacuolar or autophagy-related pathways [66,67].

PDEVs are enriched in phosphatidic acid, phytosterols, and small RNAs involved in inter-kingdom communication. Tetraspanins, including CD9, CD63, and CD81, are widely recognized as canonical EV markers and have also been identified in PDEVs. Structurally characterized by four transmembrane domains, these proteins organize membrane microdomains and regulate intracellular trafficking and signaling [68]. Although advances in isolation techniques have improved EV characterization, standardization and scalability remain challenges, as discussed below.

In mammals (Table 1), EVs comprise the following: (i) Exosomes (30–150 nm), whose formation begins with the inward budding of the endosomal membrane to form intraluminal vesicles within multivesicular bodies (MVBs). This process is regulated by mechanisms such as the endosomal sorting complex required for transport (ESCRT) machinery, referred to as the ESCRT-dependent pathway, or through ESCRT-independent mechanisms like ceramide-mediated pathways. Exosomes are characteristically enriched in CD9, CD63, and CD81, defined as tetraspanin markers. (ii) Microvesicles (50–1000 nm) formed by plasma membrane budding. They are also known as ectosomes or microparticles, and unlike exosomes, their biogenesis does not involve the endosomal trafficking system. (iii) Apoptotic bodies (1000–5000 nm) released during apoptosis. They form through membrane blebbing, where the plasma membrane fragments to encapsulate cellular debris, organelles, and nuclear material. In mammalian systems, tetraspanins additionally participate in adhesion, migration, and intercellular communication, and their differential expression in cancer underscores their diagnostic, prognostic, and therapeutic relevance [68,69].

Both PDEVs and MDEVs have been investigated in the context of chronic wounds and age-related dysfunction in tissue repair (Table 2). MDEVs, particularly those derived from mesenchymal stromal cells (MSC-EVs), are associated with immunomodulatory and regenerative effects. PDEVs have been reported to exhibit antioxidant, anti-inflammatory, and regenerative properties, along with high biocompatibility. These features have led to their consideration in the area of regenerative medicine and drug delivery, despite several limitations which impinge on improving production standardization for clinical applications [67].

### 4.1. PDEVs

PDEVs have recently gained considerable attention as a novel class of biotherapeutics and drug delivery systems, owing to their favorable biosafety profile compared with MDEVs. The absence of zoonotic pathogens, low intrinsic immunogenicity, and scalable production processes position PDEVs as highly attractive platforms for regenerative medicine and translational nanotherapy [7,90,91].

The physicochemical properties of PDEVs are strongly influenced by their source and play a central role in determining their biological behavior, particularly in relation to their interaction with tissues and ability to cross biological barriers.

Their size distribution is notably heterogeneous and source-dependent. Although most PDEVs fall within the 30–500 nm range, vesicles derived from certain plants, such as carrot, can reach sizes of up to 1500 nm (Table 3) [92]. This nanoscale dimension is relevant for their interaction with biological systems, as it can facilitate penetration across barriers such as the skin, distinguishing them from freely circulating molecules. In parallel, surface charge contributes to their colloidal stability and interaction with the extracellular environment. They generally exhibit a negative zeta potential, typically ranging from −1.5 to −49.2 mV [71], with more negative values often associated with increased stability.

Beyond these physical characteristics, the lipid composition of PDEVs represents a key determinant of their biological interactions. In contrast to MDEVs, which are enriched in cholesterol and sphingomyelin, PDEVs are predominantly composed of phospholipids such as phosphatidic acid, phosphatidylcholine, and phosphatidylethanolamine [70]. The relative abundance of these lipids varies markedly depending on the plant source and can influence cellular uptake and biodistribution. For example, ginger-derived vesicles are particularly rich in phosphatidic acid, whereas vesicles from other sources, such as orange, contain substantially lower amounts [71]. These compositional differences have been associated with distinct interaction patterns with recipient cells or microorganisms. Similarly, vesicles enriched in phosphatidylcholine, such as those derived from grapefruit, have been observed to display different distribution profiles within the body, including transport beyond the site of initial exposure [71].

On the basis of these properties, phosphatidic acid, for example, which constitutes nearly half of the lipid content in ginger-derived vesicles, mediates structural integrity and intracellular interactions, including binding to forkhead box protein A2, which is involved in cellular differentiation and tissue homeostasis. Thus, this interaction may influence how cells respond to injury, including activating repair-associated transcriptional programs [89]. Moreover, garlic-derived vesicles contain dodecanoyl-phosphatidylcholine, capable of inhibiting NLRP3 inflammasome activation, with an impact on the regulation of the pro-inflammatory environment predominant during the aging process [76].

In addition, PDEVs encapsulate a variety of plant secondary metabolites, effectively mirroring the phytochemical profile of their source. Some of them, such as gingerols, ginsenosides, aloin, and rutin, contribute to tissue tropism and therapeutic specificity [75], exerting coordinated antioxidant and anti-inflammatory effects, acting by downregulating NF-κβ, IL-6, IL-8, and TNF-α expression. In addition, in mice, it was demonstrated that PDEVs promote macrophage polarization from a pro-inflammatory M1 phenotype toward a reparative M2 phenotype, characterized by increased CD206 and IL-10 expression as well as reduced TNF-α and IL-6 production [6,77,93]. This favors a reduction in the pro-inflammatory milieu that could characterize chronic wounds and impact the proliferation and remodeling phases of the tissue repair process.

Furthermore, preclinical studies have documented broad regenerative potential of PDEVs across tissues. In cutaneous repair, ginseng-derived vesicles enhance angiogenesis in diabetic ulcers via glycolytic reprogramming [79], while aloe-derived vesicles activate Nrf2/ARE signaling to mitigate photoaging, due to promotion of nuclear translocation of Nrf2 and upregulation of cytoprotective enzymes, including heme oxygenase-1 and NQO1, thereby reducing ROS accumulation and preserving mitochondrial integrity [8,66,91]. Moreover, olive leaf-derived vesicles incorporated into hydrogels improve UV-induced skin injury repair [80,94], and grapefruit-derived vesicles reduce ROS accumulation, restore redox balance, and enhance keratinocyte proliferation and migration, facilitating wound healing [80].

In other models of regeneration, such as in bone regeneration, yam-derived vesicles activate BMP-2/p-p38 signaling [86] and tomato-derived vesicles promote chondrocyte differentiation through SOX9 and ACAN upregulation [95].

In the case of liver fibrosis, important roles can be performed by shiitake-derived vesicles, suppressing NLRP3 activation [77], and cannabis-derived vesicles, modulating the gut–liver axis [96].

Beyond intrinsic bioactivity, PDEVs function as efficient delivery vehicles. They exhibit enhanced bioavailability and cellular uptake compared with synthetic liposomes [89,97], maintain stability across broad pH ranges [75,89], and can be loaded via electroporation or freeze–thaw cycles [93]. Engineered ginger-derived vesicles loaded with survivin-targeting siRNA and functionalized with folate-targeting moieties demonstrated effective tumor suppression without detectable toxicity [88], highlighting their potential for systemic nucleic acid delivery. Furthermore, in ginger-derived EVs, miR-168c regulates the glucocorticoid-induced leucine zipper protein (TSC22D3), a key mediator of anti-inflammatory effects [87].

Innovative biomaterial integration strategies further expand therapeutic applicability. For example, injectable hydrogel systems incorporating engineered vesicles promote coordinated neurogenesis–angiogenesis crosstalk and macrophage reprogramming in diabetic wound models, accelerating repair while maintaining biocompatibility [84].

Despite promising preclinical evidence, several barriers limit clinical translation. Standardized isolation methods remain lacking [66]. Ultracentrifugation and size-exclusion chromatography (SEC) may introduce batch variability and protein contamination, and long-term storage stability remains suboptimal. Emerging strategies include artificial intelligence-guided cargo optimization, 3D bioprinting of PDEV-integrated scaffolds, and CRISPR/Cas9-mediated plant engineering to generate vesicles with defined molecular signatures [66,87,93].

Importantly, the properties of PDEVs are not solely determined by plant species and by composition characteristics but are also modulated by environmental and agricultural factors. Growth conditions, including cultivation practices, can influence the biochemical composition of the vesicles. For instance, plants grown under organic conditions have been reported to yield vesicles with higher antioxidant capacity compared to those from conventional agriculture. Additionally, environmental stresses such as drought, temperature fluctuations, or pathogen exposure can alter both the quantity and composition of vesicles produced. Even variables such as the timing of harvest or the specific plant tissue used for isolation can lead to measurable differences in vesicle composition, including shifts in lipid profiles and bioactive compound content [92].

Collectively, current evidence supports PDEVs as multifunctional, biologically active nanoplatforms capable of modulating inflammation, oxidative stress, and immune polarization, potentially contributing to regenerative medicine. Their safety profile, scalability, and engineering flexibility underscore their substantial translational potential in regenerative medicine, immunomodulation, and precision drug delivery. Moreover, the standardization of isolation methods remains an issue to be solved.

**Table 3:** Key characteristics and anti-aging applications in tissue repair of PDEVs.

Source	Size and Charge	Application for Aging-Related Tissue-Repair Dysfunction	Reference
*Panax ginseng* (Root)	Size: 344.8 nm Charge: −25.4 mV	Facilitates anti-senescence effects in human skin cells; reduces senescence-associated-*β-Gal* levels and senescence markers such as p53, p21, and p16	[98]
*Phellinus linteus* (Mushroom)	Size: <500 nm Charge: Negative	Inhibits UV-induced skin aging and cellular senescence in HaCaT cells through cross-kingdom regulation	[99]
*Malus domestica* (Apple)	Size: ~110.5 nm Charge: Negative	Reinforces the skin barrier and provides anti-aging activity in fruit calli and human dermal fibroblasts	[100]
*Aloe vera* (Peels/Gel)	Size: 90–110 nm Charge: Negative	Mitigates skin photoaging by activating the Nrf2/ARE pathway to alleviate UV-induced oxidative stress	[66]
*Olea europaea* (Leaves)	Size: <500 nm Charge: Negative	Protects against UVB-induced damage, reduces skin wrinkles, and increases collagen and elastin fibres	[87]

Note: PDEVs typically range in size from 30 to 500 nm and generally maintain a negative zeta potential often exceeding −20 mV, which contributes to their high stability.

### 4.2. MDEVs

MDEVs have emerged as key regulators of cutaneous wound healing. Accumulating evidence indicates that these EVs actively participate in all phases of the wound healing process—hemostasis, inflammation, proliferation, and remodeling—through the transfer of bioactive lipids, proteins, and nucleic acids that modulate intercellular communication within the wound microenvironment [83]. For example, as assessed above, during the hemostatic phase, PEVs, derived from platelets, the most abundant EV population in circulation, play a central role in coagulation [18]. Their procoagulant activity is primarily mediated by the surface exposure of phosphatidylserine, activation of integrin αIIbβ3, and the transfer or induction of tissue factors. In addition, PEVs may transport components of the NADPH oxidase 1 complex, thereby amplifying platelet activation and thrombus stabilization [72].

In the inflammatory phase, MDEVs exert context-dependent immunomodulatory effects. Neutrophil-derived EVs can display either pro- or anti-inflammatory properties depending on the activation state of the parental cells. Importantly, EVs released by M2 macrophages and wound-edge keratinocytes promote the transition from a pro-inflammatory M1 phenotype to a reparative M2 phenotype, a crucial step for inflammation resolution [78]. Through the delivery of specific miRNAs and proteins, EVs regulate ROS production, cytokine secretion, and the expression of inflammation-related genes, thereby shaping the inflammatory microenvironment.

During the proliferative phase of tissue repair, MDEVs play a central role in coordinating intercellular communication, thereby supporting the structural and functional restoration of damaged tissue. At this stage, their activity is closely associated with the promotion of angiogenesis, the regulation of fibroblast function, and the integration of signaling between multiple cell populations involved in healing.

MDEVs can transfer bioactive molecules, including pro-angiogenic miRNAs such as miRNA-126 and specific proteins that enhance endothelial cell migration and tube formation [74]. These processes are further supported by the activation of intracellular signaling cascades, including pathways such as PI3K/Akt/GLUT4 and AKT/GSK3β, which contribute to endothelial cell survival and proliferation [82,96].

Concurrently, MDEVs regulate fibroblast activity, a fundamental component of the proliferative phase, given the role of these cells in ECM deposition and granulation tissue formation. Through the delivery of regulatory miRNAs, including miR-21 and miR-146a, these EVs can influence fibroblast proliferation, migration, and collagen synthesis [74].

The effectiveness of the proliferative phase also depends on the coordinated interaction among keratinocytes, fibroblasts, endothelial cells, and immune cells, and MDEVs are integral to this crosstalk. They mediate signaling exchanges that regulate processes such as re-epithelialization and basement membrane restoration, contributing to the re-establishment of tissue integrity [26]. At the same time, MDEVs are involved in shaping the immune environment, particularly by influencing macrophage polarization. The transition from a pro-inflammatory (M1) to a pro-reparative (M2) phenotype is a critical step in resolving inflammation and promoting tissue regeneration, and EV-mediated signaling has been implicated in facilitating this shift [73,78]. For instance, miR-146a acts as a negative regulator of the toll-like receptor (TLR) 4 signaling pathway, effectively dampening pro-inflammatory signals and pushing the macrophage toward an anti-inflammatory M2 state.

In the remodeling phase, MDEVs regulate ECM deposition and scar formation. Fibroblast- and endothelial cell-derived EVs modulate collagen synthesis and crosslinking and promote fibroblast differentiation into myofibroblasts via miR-21, α-SMA, and N-cadherin signaling. Moreover, MDEVs enriched in HSP90α, STAT3, and pro-regenerative miRNAs contribute to wound contraction and matrix reorganization [83].

Among the various MDEV sources, MSC-EVs are the most extensively investigated in regenerative medicine. In the hemostatic phase, MSC-EVs contain both pro- and anticoagulant mediators and can activate both intrinsic and extrinsic coagulation pathways. MSC-EVs exert pronounced anti-inflammatory effects by ROS and apoptosis, promoting M2 macrophage polarization through several miRNAs (e.g., miR-223, miR-146a, miR-34a, and miR-124), and modulating signaling pathways such as PTEN/AKT and TLR 4/NF-κB [73]. These mechanisms lead to decreased levels of pro-inflammatory cytokines (TNF-α, IL-6, IL-8) and increased expression of anti-inflammatory mediators such as IL-10. In the proliferative phase, MSC-EVs enhance fibroblast migration and proliferation, stimulate the synthesis of ECM components, and promote angiogenesis via mediators including miRNA and growth factors [74]. During tissue remodeling, bone marrow-derived MSC-EVs have been shown to increase collagen I deposition, α-SMA expression, and the formation of skin appendages, whereas adipose-derived MSC-EVs appear to attenuate excessive fibrosis and favor a more physiological scar architecture.

Overall, EVs, both endogenous and MSC-derived, constitute a complex and highly versatile communication system that orchestrates the dynamic cellular and molecular events underlying cutaneous wound repair. Their ability to deliver functional miRNAs, proteins, and lipids in a cell-free format positions them as promising candidates for next-generation regenerative therapies, particularly in the treatment of chronic wounds, burns, and inflammatory skin disorders.

Table 4 provides a summary of the potential applications of MDEVs in aging-related tissue-repair dysfunction.

## 5. Future Perspectives and Conclusions

Aging is characterized by the progressive decline of multiple physiological systems, driven by the interconnected hallmarks of aging, including cellular senescence, stem cell exhaustion, genomic instability, mitochondrial dysfunction, and chronic low-grade inflammation [12]. This systemic deterioration profoundly compromises tissue repair capacity. In older individuals, impaired immune responsiveness, defective macrophage polarization, reduced stem/progenitor cell activity, altered mitochondrial bioenergetics, and ECM disorganization collectively delay wound closure and favor maladaptive remodeling. Persistent inflammatory signaling, dysfunctional intercellular communication, and impaired resolution pathways contribute to chronic wound states and fibrotic sequelae. Clinically, these mechanisms underline highly prevalent and disabling conditions such as chronic venous and diabetic ulcers, as well as progressive fibrotic disorders including hepatic fibrosis.

Within this framework, MDEVs have particularly emerged as key regulators of the aging–repair axis. MDEVs participate in all phases of tissue regeneration by modulating inflammatory cascades, oxidative stress responses, angiogenesis, fibroblast activation, and ECM remodeling. MSC-EVs exert potent immunomodulatory and pro-regenerative effects by promoting macrophage polarization toward a reparative M2 phenotype, attenuating pro-inflammatory cytokine production, enhancing collagen deposition, and stimulating neovascularization. These activities directly counteract central aging-associated dysfunctions, including inflammaging, impaired angiogenic competence, and defective matrix turnover. Beyond their immunomodulatory role, MDEVs restore age-disrupted intercellular communication through the horizontal transfer of regulatory miRNAs, proteins, lipids, and bioactive metabolites. By modulating pathways involved in oxidative stress control, mitochondrial homeostasis, apoptosis resistance, and tissue remodeling, MDEVs recalibrate the inflammatory–proliferative balance required for effective regeneration. Their cell-free nature further strengthens their translational appeal, offering a safer, more controllable, and potentially more scalable alternative to stem cell transplantation, particularly in older patients with comorbidities.

Accordingly, EVs can be considered natural and innovative tools in regenerative medicine, with applications ranging from cutaneous wound healing to protection of internal organs and regeneration of complex tissues such as bone and cartilage.

Beyond systemic therapeutic applications, EVs hold substantial promise in dermatology and cosmetic science, particularly PDEVs. Cutaneous aging is characterized by collagen fragmentation, elastin degradation, reduced fibroblast activity, oxidative stress accumulation, and immune imbalance. EV-based formulations and PDEV applications have demonstrated the capacity to stimulate dermal fibroblast proliferation, enhance collagen types I and III synthesis, promote angiogenesis, and attenuate inflammatory signaling.

Despite these properties of EVs—advanced bioactive components for next-generation cosmeceuticals and regenerative aesthetic interventions—their clinical translation, encompassing both MDEVs and PDEVs, remains constrained by a series of interrelated challenges that must be systematically addressed to enable large-scale biomedical application.

A major limitation lies in the lack of standardized isolation and purification protocols. EVs are highly sensitive to methodological variations, and even minor differences in procedures such as ultracentrifugation, filtration, or SEC can markedly influence vesicle yield, purity, and biological activity. At present, no universally accepted approach ensures both high recovery and high purity, resulting in significant variability across studies and limiting reproducibility, particularly in the case of PDEVs [87,103]. Commonly used methods are also prone to batch-to-batch inconsistency and co-isolation of contaminants, including proteins and other extracellular components. This issue is compounded by the absence of consensus in EV nomenclature and classification, particularly for PDEVs, where terms such as “plant exosomes” and “plant-derived nanoparticles” are often used interchangeably, reflecting broader gaps in methodological and conceptual harmonization [87,103].

Closely linked to this is the intrinsic heterogeneity of EV populations. Both PDEVs and MDEVs comprise a diverse array of vesicles that differ in size, morphology, membrane composition, and molecular cargo. Current isolation techniques are largely non-selective and do not allow for the enrichment of homogeneous subpopulations. As a result, even engineered or vesicle-mimetic systems may display substantial variability in physicochemical properties and cargo content. This heterogeneity complicates the identification of functionally relevant vesicle subsets, the establishment of precise dosing regimens, and the reproducibility of therapeutic outcomes, ultimately limiting clinical reliability [104].

Challenges in characterization and traceability further impact the field, particularly for PDEVs. While MDEVs can be identified using relatively well-established protein markers such as tetraspanins, PDEVs lack universally accepted molecular signatures, making their validation less straightforward. Moreover, PDEVs are highly influenced by biological and environmental variables, including species, geographical origin, developmental stage, and harvesting conditions. These factors can significantly alter vesicle composition and bioactive cargo, underscoring the need for rigorous reporting standards and more comprehensive characterization frameworks [92].

Another critical bottleneck concerns scalability and manufacturing. Although plant sources offer advantages in terms of availability and sustainability, current extraction and purification approaches for both PDEVs and MDEVs are labor-intensive, time-consuming, and not readily adaptable to industrial-scale production. The generation of clinical-grade EVs requires strict quality control and compliance with Good Manufacturing Practice standards, which remain difficult to achieve with existing methodologies. Consequently, the development of automated, scalable, and standardized bioprocessing platforms is essential for advancing EV-based applications toward clinical use [71].

In addition, the biological behavior of EVs *in vivo* is not yet fully understood. While increasing evidence supports their role in intercellular and even cross-kingdom communication, the mechanisms governing cargo loading, biodistribution, cellular uptake, and tissue-specific targeting remain incompletely defined. This knowledge gap restricts the ability to design EV-based therapies with predictable efficacy and safety profiles and raises concerns regarding potential off-target effects [87,94].

Safety considerations further complicate clinical translation. Although EVs are generally considered to exhibit low immunogenicity, their capacity to carry bioactive molecules introduces potential risks, including pro-tumorigenic signaling, unintended immune modulation, and off-target biological effects. Moreover, long-term toxicity remains insufficiently characterized, particularly in the context of repeated or chronic administration, where bioaccumulation or delayed adverse effects cannot yet be excluded.

Finally, regulatory challenges constitute a significant obstacle. EV-based therapeutics occupy a complex and still evolving regulatory landscape, sharing features with biologics, cell-based therapies, and drug delivery systems. Current frameworks remain fragmented and lack harmonization across jurisdictions, while standardized criteria for EV identity, purity, potency, and safety are still under development. Existing guidelines, such as MISEV, provide an important foundation but are not yet fully adapted to account for the specific characteristics of plant-derived vesicles, highlighting the need for more tailored regulatory pathways [71,92].

In summary, although EVs derived from both plant and mammalian sources are being extensively investigated in the context of drug delivery and regenerative medicine, their translation into clinical practice is limited by a combination of technical, biological, and regulatory challenges that require coordinated and systematic resolution [105].

Addressing these challenges through coordinated efforts in standardization, mechanistic understanding, scalable manufacturing, and regulatory alignment will be essential to unlock their full therapeutic potential as a versatile and transformative platform at the intersection of regenerative medicine, geroscience, and aesthetic dermatology. Continued interdisciplinary research integrating molecular gerontology, biomaterials engineering, and translational dermatology will be crucial to unlock their therapeutic and commercial potential fully.

## Figures and Tables

**Table 1:** Summary of MDEV types and characteristics.

EV Type	Size Range	Cellular Origin and Biogenesis	Key Mechanisms and Markers	Reference
Exosomes	30–150 nm	Endosomal pathway; maturation of MVBs and fusion with plasma membrane	ESCRT-dependent or ceramide-mediated; enriched in CD9, CD63, and CD81	[69,70,71]
Microvesicles	50–1000 nm	Direct outward budding or “shedding” from the plasma membrane	Cytoskeletal rearrangement and membrane protrusion	[70,71]
Apoptotic bodies	1000–5000 nm	Plasma membrane fragmentation during apoptosis	Membrane blebbing; encapsulates nuclear fragments and organelles	[71]

**Table 2:** Summary of EVs in tissue repair and associated pathologies.

Phase or Repair Process	Vesicle Type	Mechanism/ Biological Effect	References
Hemostasis	PEVs; MSC-EVs	Modulation of platelet function via superoxide generation; procoagulant activity in adipose-derived MSCs	[72,73,74]
Inflammation	Ginger, garlic, and garlic EVs; exosomes	Promotion of M1 to M2 macrophage phenotypic switch; inhibition of the NLRP3 inflammatory effects	[75,76,77,78]
Proliferation phase	PDEVs (aloe, grapefruit, mung bean); MSC-EVs	Glycolysis reprogramming to stimulate angiogenesis; activation of signaling pathways (AKT/GSK3β, PI3K/Akt/GLUT4).	[79,80,81,82]
Remodeling phase	Cargo-specific EVs (micro-RNA (miRNAs))	Regulation of fibroblast activation and collagen production via miR-21, miR-146a, and miR-126; ECM remodeling	[83]
Chronic wounds (e.g., diabetic ulcers)	PDEVs and hybrid repair systems	Restoration of neurogenesis–angiogenesis crosstalk; promotion of macrophage reprogramming to close non-healing wounds	[36,79,84]
Fibrosis (liver and skin)	Therapeutic EVs; senescent HSCs	Targeting senescent HSCs to reduce excessive fibrin/collagen deposition; modulation of fibrogenesis	[47,49,51]
Aging and inflammaging	Plant-derived nanovesicles (aloe vera, carrot, yam)	Mitigation of photoaging via nuclear factor erythroid 2-related factor 2 (Nrf2)/antioxidant responsive element (ARE) pathway; antioxidant effects; protection of intestinal and skin barriers	[66,85,86,87]
Malignant transformation (cancer)	Tumor-derived and PDEVs	Use of nanovectors (e.g., ginger or grapefruit) for systemic drug/small interfering RNA (siRNA) delivery; cancer viewed as a “wound that does not heal”	[59,62,88,89]

**Table 4:** Key characteristics and anti-aging applications of MDEVs.

Source	EV Size and Charge	Application in Aging-Related Tissue-Repair Dysfunction	Reference
PEVs	30–100 nm; 100 nm–1 μm	Mediate intercellular signaling through their cargo of growth factors, miRNAs, and cytokines; stimulate cell proliferation, migration, and extracellular matrix production	[72,101]
MSCs	Size: 40–120 nm Charge: Negative	Protect against oxidative stress-induced skin injury and stimulate tissue regeneration	[94]
Induced Pluripotent Stem Cells	Size: 40–120 nm Charge: Negative	Exosomes that improve the aging process of human skin fibroblasts	[102]

## Data Availability

No new data were created or analyzed in this study.

## Abbreviations

The following abbreviations are used in this manuscript:

ECM	extracellular matrix
EVs	extracellular vesicles
PDEVs	plant-derived extracellular vesicles
MDEVs	mammalian-derived extracellular vesicles
GP	glycoprotein
PEVs	platelet-derived extracellular vesicles
ROS	reactive oxygen species
NETs	neutrophil extracellular traps
TNF	tumour necrosis factor
IL	interleukin
NO	nitric oxide
Th	T helper cells
TGF	transforming growth factor
VEGF	vascular endothelial growth factor
Tregs	regulatory T cells
IFN	interferon
PDGF	platelet-derived growth factor
FGF	fibroblast growth factor
KGF	keratinocyte growth factor
MMPs	metalloproteases
SASP	senescence-associated secretory phenotype
NF-κB	nuclear factor kappa-light-chain-enhancer of activated B cells
mTOR	mammalian target of rapamycin
VLUs	venous leg ulcers
DFUs	diabetic foot ulcers
SCC	squamous cell carcinoma
TβR2	TGF-β type II receptor
TβR1	TGF-β type I receptor
HSCs	hepatic stellate cells
NK	natural killer cells
NKT	NK T cells
WHFC	wound healing–fibrosis–cancer
MU	Marjolin’s ulcer
BCC	basal cell carcinoma
EMT	epithelial–mesenchymal transition
MVBs	multivesicular bodies
Nrf2	nuclear factor erythroid 2-related factor 2
ARE	antioxidant responsive element
ESCRT	Endosomal Sorting Complex Required for Transport
MSC-EVs	mesenchymal stem cell-derived EVs
miRNA	microRNA
siRNA	small interfering RNA
TET	tetraspanin
SEC	size-exclusion chromatography
TLR	toll-like receptor
